# Assessing the Impact of ChatGPT in Dermatology: A Comprehensive Rapid Review

**DOI:** 10.3390/jcm13195909

**Published:** 2024-10-03

**Authors:** Polat Goktas, Andrzej Grzybowski

**Affiliations:** 1 UCD School of Computer Science, University College Dublin, D04 V1W8 Dublin, Ireland; polat.goktas@ucd.ie; 2 Department of Ophthalmology, University of Warmia and Mazury, 10-719 Olsztyn, Poland; 3 Institute for Research in Ophthalmology, Foundation for Ophthalmology Development, 61-553 Poznan, Poland

**Keywords:** artificial intelligence, ChatGPT, dermatology, ethics, medical writing, natural language processing, large language model, patient education, teledermatology

## Abstract

**Background/Objectives**: The use of artificial intelligence (AI) in dermatology is expanding rapidly, with ChatGPT, a large language model (LLM) from OpenAI, showing promise in patient education, clinical decision-making, and teledermatology. Despite its potential, the ethical, clinical, and practical implications of its application remain insufficiently explored. This study aims to evaluate the effectiveness, challenges, and future prospects of ChatGPT in dermatology, focusing on clinical applications, patient interactions, and medical writing. ChatGPT was selected due to its broad adoption, extensive validation, and strong performance in dermatology-related tasks. **Methods**: A thorough literature review was conducted, focusing on publications related to ChatGPT and dermatology. The search included articles in English from November 2022 to August 2024, as this period captures the most recent developments following the launch of ChatGPT in November 2022, ensuring that the review includes the latest advancements and discussions on its role in dermatology. Studies were chosen based on their relevance to clinical applications, patient interactions, and ethical issues. Descriptive metrics, such as average accuracy scores and reliability percentages, were used to summarize study characteristics, and key findings were analyzed. **Results**: ChatGPT has shown significant potential in passing dermatology specialty exams and providing reliable responses to patient queries, especially for common dermatological conditions. However, it faces limitations in diagnosing complex cases like cutaneous neoplasms, and concerns about the accuracy and completeness of its information persist. Ethical issues, including data privacy, algorithmic bias, and the need for transparent guidelines, were identified as critical challenges. **Conclusions**: While ChatGPT has the potential to significantly enhance dermatological practice, particularly in patient education and teledermatology, its integration must be cautious, addressing ethical concerns and complementing, rather than replacing, dermatologist expertise. Future research should refine ChatGPT’s diagnostic capabilities, mitigate biases, and develop comprehensive clinical guidelines.

## 1. Introduction

The integration of artificial intelligence (AI) into dermatology marks a significant advancement in medical education and practice, offering transformative capabilities that range from diagnostic accuracy to enhanced patient interaction. Among the many AI tools being explored, ChatGPT, an advanced language model developed by OpenAI and first released on 30 November 2022, has gained prominence as a key tool in various fields, including healthcare [1,2]. It utilizes a deep learning model to generate human-like conversational responses and enables users to refine and steer the conversation towards a desired length, format, style, level of detail, and language [3,4,5,6]. This study focuses on ChatGPT due to its widespread adoption, extensive validation in medical contexts, and superior performance in dermatological tasks compared to other large language models (LLMs).

ChatGPT’s growing prominence in healthcare, particularly in dermatology, is driven by several factors. Firstly, ChatGPT’s widespread adoption and accessibility make it an ideal candidate for exploring AI applications in healthcare, providing a comprehensive examination of its impact and utility [7]. Secondly, extensive research has validated ChatGPT’s utility in medical contexts, with studies demonstrating its ability to pass dermatology certification exams, provide reliable patient education, and assist in medical writing [8,9]. Thirdly, ChatGPT has shown significant advancements in handling medical queries, outperforming some contemporary LLM models in specific healthcare tasks [10,11]. Additionally, the specific applications of ChatGPT in dermatology—ranging from patient education to clinical decision support—underline its suitability for this review. Finally, ChatGPT’s prominence in discussions about AI ethics makes it a particularly relevant example for addressing the ethical and legal challenges of AI in dermatology [12].

Dermatology, a specialty heavily reliant on visual assessments, patient communication, and complex decision-making, presents both opportunities and challenges for the integration of AI tools like ChatGPT [13]. While AI technologies, particularly in diagnostic imaging and disease classification, have been at the forefront of dermatological advancements, the application of LLMs such as ChatGPT in this field remains relatively new and underexplored [14,15,16]. This raises the following critical question: *Is ChatGPT a genuinely useful tool in dermatology, or does it merely contribute to the growing technological hype* [2,17]?

The potential utility of ChatGPT in dermatology extends beyond diagnostic imaging. Recent studies have highlighted ChatGPT’s ability to engage in patient education, provide clinical decision support, and even assist in medical writing. For instance, Dunn et al. (2023) [18] found that AI-generated dermatology case reports were almost indistinguishable from those written by humans, with AI scoring higher in writing conventions and logical sequencing. However, inaccuracies in references and cutaneous findings highlighted limitations in precision. Shapiro et al. (2024) [19] explored AI-driven anamnesis and diagnosis in teledermatology, showing that a GPT-based chatbot could accurately diagnose conditions like alopecia areata, but emphasized the need for more relevant questioning and integration with electronic medical records to enhance consultation accuracy.

**Figure 1:**
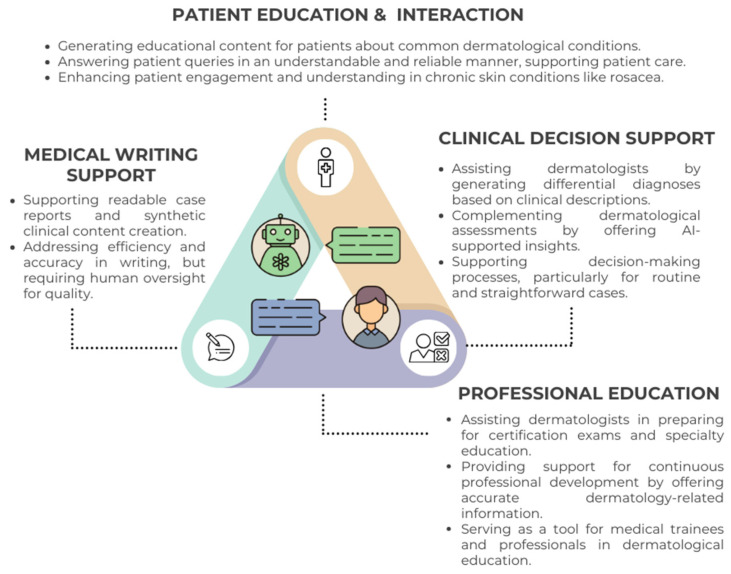
Overview of ChatGPT’s core applications in dermatology, highlighting its roles in patient education and interaction, professional education, clinical decision, and medical writing supports.

ChatGPT has also been tested for generating patient-facing content about common dermatological conditions like rosacea and melanoma. Young et al. (2023) [20] assessed ChatGPT’s responses to melanoma-related queries, finding a mean accuracy score of 4.88/5, with 92% of the content deemed suitable for patient education. However, only 64% of the responses were deemed clinically sufficient, often lacking crucial details like the frequency of follow-up skin exams. Yan et al. (2024) [21] further evaluated ChatGPT’s responses to rosacea queries, showing high reliability percentages (92.22–97.78%) and clinical applicability percentages (98.61–100.00%) across various treatment categories, supporting its effectiveness in patient education for chronic conditions like rosacea, though human oversight was recommended for managing complex cases. Additionally, ChatGPT has demonstrated potential in supporting dermatologists by generating differential diagnoses based on clinical descriptions [22]. These applications suggest that ChatGPT could serve as a valuable adjunct to traditional dermatological practice, particularly in settings where patient interaction and education are critical [23,24]. Figure 1 illustrates the key applications of ChatGPT in dermatology, including patient education, clinical decision support, and medical writing.

Despite these promising applications, significant limitations persist. ChatGPT’s diagnostic accuracy, particularly in identifying malignant skin lesions, remains inconsistent. Studies comparing ChatGPT to other AI models, such as Claude 3 Opus, have shown that, while these tools can assist in providing differential diagnoses, they still struggle with the nuanced and often subjective nature of dermatological assessments [13,25]. For example, ChatGPT’s performance in diagnosing melanoma based on physical exam descriptors or dermoscopic images has been found lacking, with accuracy rates that raise concerns about its reliability in clinical practice [26]. These findings underline the need for a more cautious approach to integrating ChatGPT into dermatological workflows, ensuring that it complements rather than replaces the expertise of trained clinicians [6,27,28].

Furthermore, the ethical and legal implications of using AI in dermatology cannot be overlooked. The rapid deployment of AI tools like ChatGPT raises questions about data privacy, informed consent, and the potential for bias in AI-generated recommendations. As noted by Khan et al. (2024), the integration of AI into clinical practice must be guided by stringent ethical standards to prevent the exacerbation of existing health disparities and ensure equitable care [6]. The lack of transparency in the data used to train models like ChatGPT also poses a significant challenge, as inaccurate or biased outputs could lead to harmful clinical decisions [23,28,29]. These concerns highlight the importance of developing robust regulatory frameworks and establishing clear guidelines for the ethical use of AI in dermatology [17,30].

In addition to its clinical applications, ChatGPT’s role in medical writing represents another area of interest. The ability of ChatGPT to generate high-quality, readable case reports indistinguishable from those written by human authors has sparked both excitement and concern within the medical community. While this capability could streamline the writing process and enhance productivity, it also raises questions about the potential for AI to depersonalize patient care and diminish the value of human expertise in scientific communication [18,31]. Moreover, the risk of AI-generated misinformation, a phenomenon known as the “*hallucination*” effect, further complicates the use of ChatGPT in academic and clinical settings [32].

Therefore, this rapid review aims to contribute to the growing body of literature by thoroughly analyzing ChatGPT’s role in dermatology. We examine its applications in clinical diagnostics, patient education, and medical writing while also addressing the ethical and legal challenges that accompany its use. By reviewing the existing research and identifying gaps, we seek to clarify ChatGPT’s potential advantages and limitations within dermatology. Our ultimate goal is to provide a balanced view on integrating this AI tool into dermatological practice, ensuring it supports rather than detracts from patient care. As AI evolves, it is essential to remain vigilant about its impacts, ensuring that technological progress leads to real improvements in patient outcomes [24,33].

## 2. Materials and Methods

### 2.1. Study Design

This rapid review focuses on critically evaluating the role of ChatGPT in dermatology by analyzing the existing literature on its applications in areas such as clinical diagnostics, patient education, and medical writing. Additionally, it addresses the ethical, legal, and regulatory challenges associated with its use. To provide a comprehensive overview, this study also provides ChatGPT’s performance with other LLM models in dermatology, such as GPT-4, emphasizing the implications for both future research and clinical practice.

### 2.2. Search Strategy

We conducted a comprehensive search of Web of Science, Scopus, PubMed, and MEDLINE between June and August 2024 to identify studies related to LLM and dermatology, with a specific emphasis on ChatGPT. The search was limited to articles published in English from November 2022 to August 2024 because ChatGPT was launched on 30 November 2022 [1] and the literature relevant to its application in dermatology would be from that date onward. The search strategy was developed in collaboration with an experienced medical librarian to ensure that it was both comprehensive and precise.

Key search terms included the following:

- “ChatGPT” AND “dermatology”;
- “large language models” AND “natural language processing” AND “AI in dermatology”;
- “ethical considerations” AND “AI in healthcare”.

Boolean operators were used to refine the search results, which were limited to English published articles from November 2022 to August 2024 to ensure relevance and inclusion of recent advancements. The reference lists of the included articles were manually reviewed to identify additional relevant studies not captured by the database search.

### 2.3. Inclusion and Exclusion Criteria

Studies were eligible for inclusion if they met the specific criteria. These criteria included original research articles, systematic reviews, meta-analyses, narrative reviews, case reports, and clinical trials that focused on LLM models in dermatology, particularly ChatGPT. The studies needed to explore areas such as diagnostic capabilities, patient education roles, or medical writing applications of ChatGPT or other variants in dermatology. Furthermore, the studies had to involve dermatological patients or practitioners utilizing AI tools, and the outcomes had to address topics like diagnostic accuracy, patient outcomes, educational impacts, ethical concerns, or legal implications related to LLM use in dermatology.

The exclusion criteria applied to studies that did not specifically address AI applications in dermatology as well as opinion pieces, editorials, conference abstracts, and non-peer-reviewed articles. Studies published in languages other than English and those focusing on AI applications outside of dermatology were also excluded from the review.

### 2.4. Data Collection

All identified references were imported into EndNote X9, where duplicates were automatically removed. One independent reviewer screened the titles and abstracts of the remaining articles for eligibility. Discrepancies in inclusion decisions were resolved through discussion, and if necessary, another reviewer provided the final decision. Full-text versions of potentially relevant studies were retrieved and further assessed for eligibility. For each included study, data were extracted using a standardized data extraction strategy in Excel form. It captured details such as study design, LLM technology or model used (e.g., GPT-3.5, -4 modules), specific dermatological application (e.g., diagnosis, patient education), key findings, and any reported ethical or legal concerns.

### 2.5. Data Synthesis and Analysis

The findings from the selected studies were synthesized narratively, given the heterogeneity of the included studies. The synthesis focused on several key themes: (1) the diagnostic accuracy of ChatGPT and similar models in dermatology, particularly in identifying skin lesions and generating differential diagnoses; (2) the utility of ChatGPT in patient communication and education; (3) the role of ChatGPT in medical writing, including case reports and scientific publications; and (4) the ethical, legal, and regulatory considerations associated with the deployment of AI in dermatological practice.

Descriptive metrics provided in the included papers, including metrics such as accuracy, reliability, and clinical applicability, were used to summarize the study characteristics, and key findings were organized thematically. Where applicable, comparative analyses between ChatGPT and other AI models were highlighted to provide insights into the relative performance and limitations of different technologies in dermatology.

### 2.6. Ethical Considerations

As this study involved a review of existing literature, no new data were collected, and no ethical approval was required. However, ethical principles were adhered to throughout the review process, including maintaining objectivity, transparency, and rigor in data collection, analysis, and reporting. This study also addresses the ethical implications of integrating LLM into dermatological practice, with a focus on patient rights, privacy, and the potential impact on health disparities.

### 2.7. Limitations

This rapid review has several limitations. First, this study is subject to publication bias, as it primarily included peer-reviewed articles, which may favor studies with positive findings. Second, the exclusion of non-English publications could result in the omission of relevant studies from non-English-speaking regions. Third, the rapidly evolving nature of AI technologies may lead to the exclusion of recently developed tools and findings. Lastly, the heterogeneity of study designs and outcomes across the included articles limited the ability to perform a meta-analysis; thus, the results are presented in a descriptive manner.

## 3. ChatGPT in Dermatology Practice

Building on the advancements in AI discussed in the previous section, the integration of LLM tools such as ChatGPT into dermatology practice marks a significant step forward in clinical and educational applications. ChatGPT, a large language model developed by OpenAI, has shown promise in various medical disciplines, including dermatology, where its potential to aid in diagnosis, patient education, and treatment planning is being currently explored.

### 3.1. ChatGPT’s Performance in Dermatology Certification Exams

The capabilities of ChatGPT in a clinical context have been evaluated through its performance on dermatology-specific certification exams. In one study, ChatGPT-4 achieved a passing score on the Specialty Certificate Examination in Dermatology, significantly outperforming its predecessor, ChatGPT-3.5. While ChatGPT-3.5 scored 63%, ChatGPT-4 reached 90%, surpassing the typical pass mark of 70–72% [34]. Similarly, when tested on a practice dermatology board certification examination, ChatGPT-3.5 achieved an accuracy of 59.3%. The study highlighted that, while the model performed well in categories like basic science and pediatric dermatology, it struggled with more complex diagnoses, such as benign and malignant neoplasms [35]. These results underline the potential of ChatGPT as a supportive tool in dermatological education and certification preparation. However, they also reveal limitations in the model’s ability to handle complex, nuanced clinical cases without human oversight.

In addition, comparative analyses between ChatGPT and other AI models, such as GPT-3.5 and GPT-4, have highlighted the progressive improvements in AI’s ability to understand and respond to dermatological queries. For example, in a study evaluating ChatGPT’s performance on the Spanish medical residency entrance examination, GPT-4 outperformed GPT-3.5, demonstrating its growing competency in handling diverse medical queries across multiple specialties, including dermatology [36]. The results of these studies are summarized in Table 1, which highlights ChatGPT’s performance across various dermatology certification examinations, demonstrating its strengths and limitations in supporting clinical education.

### 3.2. Reliability and Clinical Applicability in Patient Queries

Beyond its role in professional education, ChatGPT’s utility in addressing patient queries has also been assessed. For instance, Yan et al. (2023) [21] assessed ChatGPT’s responses to common patient questions about rosacea. The model demonstrated high reliability, with accuracy rates ranging from 92.22% to 97.78% and clinical applicability between 98.61% and 100.00%. The AI’s answers closely aligned with clinical guidelines, making it a valuable tool for patient education in managing chronic skin conditions like rosacea. Similarly, Young et al. (2023) [20] evaluated ChatGPT’s responses to melanoma-related inquiries. While the model provided accurate information suitable for general patient education—achieving an accuracy score of 4.88 out of 5—only 64% of the responses were deemed adequate for clinical use. The limitations were primarily due to a lack of detailed specificity required for medical advice, highlighting the necessity of human oversight in complex cases.

Further extending the scope, a comprehensive study by Ferreira et al. (2023) assessed ChatGPT’s responses to common questions from patients about six prevalent skin conditions: acne, atopic dermatitis, alopecia, psoriasis, rosacea, and skin cancer [37]. The model achieved an overall 88% appropriateness rate, with 82 out of 93 responses graded as appropriate by board-certified dermatologists. For example, ChatGPT was found to deliver 100% appropriate responses to queries about acne and rosacea, suggesting its strong alignment with clinical standards in these areas. In contrast, the model’s performance was less robust in complex scenarios, such as responding to patient queries about alopecia and atopic dermatitis, where a significant percentage of responses (up to 25%) were deemed inappropriate due to incomplete or potentially misleading information. This underscores the need for caution when relying on AI tools in patient education, particularly for conditions that involve varied etiologies and treatment options, such as alopecia.

Moreover, Trager et al. (2023) evaluated ChatGPT’s responses to patient queries concerning basal cell carcinoma (BCC) [38]. They found that, while 84% of responses were appropriate, 16% were inappropriate due to inaccuracies or misleading information, such as suggesting that untreated BCC could progress into melanoma, a statement all reviewers rated as inaccurate. Other inappropriate responses involved subtle misrepresentations, such as the description of BCC on the ear that failed to capture its typical clinical presentation as a pearly white papule. Despite these issues, ChatGPT’s overall performance suggests that it has potential as a supplementary resource, especially in providing basic information about the diagnosis, risk factors, and treatment options for BCC.

As outlined in Table 2, these findings suggest that ChatGPT demonstrates a high degree of reliability and applicability for common dermatological patient queries. However, its limitations in providing detailed clinical advice—especially for more complex or nuanced conditions—indicate that it should be used to supplement, rather than replace, professional medical consultations.

### 3.3. Challenges in Clinical Practice and User Experience

Despite its promising applications, the use of ChatGPT in dermatology is not without challenges. A study examining ChatGPT’s capabilities in dermato-oncology, particularly in relation to actinic keratosis, found that, while the model provided accurate information for patient education, it struggled with clinical aspects such as diagnosis and treatment planning. The responses were often verbose and occasionally contained inaccuracies presented with undue confidence, which could mislead users [40]. Furthermore, a user experience evaluation revealed that, while dermatologists found ChatGPT to be an attractive and efficient tool, its clarity and accuracy were lacking, particularly in complex clinical scenarios. This suggests that, while ChatGPT can streamline information retrieval, it requires further refinement to meet the stringent demands of clinical practice.

## 4. ChatGPT and Patient Interaction

The integration of LLM, particularly ChatGPT, into dermatological practice is not only reshaping clinical diagnostics and treatment planning but also transforming patient interaction and education. As patients increasingly seek information online, tools like ChatGPT are becoming essential in bridging the gap between patients and healthcare providers. This section explores the role of ChatGPT in enhancing patient interaction, particularly in the context of dermatology, where clear communication and accurate information are crucial for effective disease management.

### 4.1. ChatGPT for Patient Education in Dermatology

Patient education is a critical component of managing dermatological conditions, where understanding disease processes and treatment options significantly impacts patient outcomes. In a pilot study by Mondal et al. (2023), ChatGPT was evaluated for its effectiveness in generating patient education materials for common dermatological conditions [39]. The study found that ChatGPT could generate easily understandable educational content suitable for high school or early college students, with an average readability score of 46.94. However, the study also noted a higher-than-expected text similarity index, indicating the potential for redundancy or lack of originality in the generated content. This highlights the need for healthcare providers to carefully review and modify AI-generated educational materials to ensure both accuracy and uniqueness.

### 4.2. Improving Patient Communication

Effective communication between patients and dermatologists is essential for ensuring that patients fully understand their condition and treatment options. ChatGPT has shown potential in facilitating this communication by generating responses to patient inquiries that are both informative and accessible. For instance, a study assessing ChatGPT’s responses to common patient questions about rosacea found that the AI demonstrated high reliability and clinical applicability, with scores ranging from 92.22% to 97.78% for reliability and 98.61% to 100.00% for clinical applicability [21]. This indicates that ChatGPT can effectively support patient education by providing accurate and relevant information, which can help patients make informed decisions about their care.

However, significant challenges persist. While ChatGPT can handle straightforward inquiries, it may struggle with more complex medical questions, leading to inaccuracies or oversimplified responses. In dermatology, this limitation is particularly concerning given the complexity and variability of skin conditions. Comparative analyses between ChatGPT and other AI models, such as GPT-3.5 and GPT-4, have shown that, while these models excel in generating general information, they may falter when detailed, domain-specific knowledge is required [41,42].

### 4.3. ChatGPT in Rural Healthcare Delivery

The role of ChatGPT in improving healthcare access, particularly in underserved rural areas, has been explored in studies. One commentary highlighted the potential of ChatGPT to bridge the healthcare gap in rural settings by providing timely and accurate medical information, which can be especially valuable in regions with limited access to healthcare professionals [43]. In dermatology, where specialist care may be scarce in remote areas, ChatGPT could serve as an initial point of contact, offering guidance on when to seek further medical attention.

### 4.4. Challenges in Patient Interaction

Despite its promising potential, the use of ChatGPT in patient interaction presents several significant challenges. One of the primary concerns is the phenomenon of “hallucinations”, where the AI produces incorrect or fabricated information, posing serious risks in clinical contexts [44]. Additionally, there is notable variability in the accuracy of ChatGPT’s responses, particularly as the complexity of the medical query increases. While ChatGPT can effectively handle common, straightforward patient inquiries, its accuracy and comprehensiveness decline when confronted with more complex medical issues [45]. Moreover, the ethical implications of integrating AI into patient interactions, including concerns about data privacy, bias, and the potential over-reliance on AI-generated content, necessitate careful consideration. These challenges highlight the urgent need for stringent guidelines and continuous oversight of AI applications in healthcare to ensure patient safety and uphold the integrity of medical practice [46]. The study also emphasizes the risks associated with using ChatGPT in clinical practice, particularly the dangers of misinformation, hallucinations, and the absence of critical human qualities essential for effective patient communication.

## 5. ChatGPT in Medical Writing

The integration of ChatGPT into medical writing, particularly in dermatology, represents a significant shift in how content is generated, reviewed, and disseminated within the field. As discussed in previous sections, AI technologies like ChatGPT have begun to play a more substantial role in various aspects of dermatology, including patient interaction and clinical decision-making. In the context of medical writing, ChatGPT offers numerous advantages but also presents certain challenges that require careful consideration.

One of the key applications of ChatGPT in dermatology is its potential to assist in drafting and editing manuscripts. Potestio et al. (2024) highlight that ChatGPT can perform tasks such as language revision, data analysis, and even the formulation of entire manuscripts [31]. This capability is particularly valuable in the time-intensive process of medical writing, allowing for researchers and clinicians to focus more on content and interpretation rather than the mechanics of writing. However, this raises the question of whether human authorship and critical thinking might be compromised as reliance on AI increases.

However, there are ongoing debates about the role of AI in medical writing, particularly concerning the quality and originality of the content generated by ChatGPT. Comparative studies have shown that, while ChatGPT can produce coherent and well-structured text, the quality of AI-generated content often falls short when compared to that written by experienced dermatologists, especially in terms of readability, empathy, and thoroughness [47]. This discrepancy highlights the limitations of ChatGPT in capturing the nuanced and context-specific knowledge that is often required in medical writing.

Moreover, the involvement of AI in scientific writing raises ethical concerns, particularly regarding authorship and the integrity of the research process. A scoping review of publications involving AI and ChatGPT revealed that, while these tools were increasingly being used in manuscript preparation, they were not listed as authors, reflecting the ongoing uncertainty about how to appropriately credit AI’s contributions in academic work [48]. This ambiguity extends to the peer review process, where AI models like ChatGPT have been explored as tools to assist in manuscript evaluation. While these tools can enhance efficiency, their effectiveness compared to human reviewers remains limited, particularly in more complex and subjective aspects of the review process [49]. This suggests that, while ChatGPT can be a helpful tool in the review process, it cannot yet fully replace human expertise.

The growing use of AI in medical writing underlines the need for clear guidelines and best practices to ensure that these technologies are used responsibly. Descriptive metrics from the studies discussed above reveal a consistent theme: while ChatGPT excels in generating structured, readable content, it often falls short in areas requiring deep domain-specific knowledge or emotional intelligence. Establishing such standards could be crucial in maintaining the integrity of medical literature while leveraging the benefits that AI can offer. As AI continues to evolve, its role in medical writing would likely expand, but this must be balanced with a commitment to upholding the quality and ethical standards of academic publishing [50].

## 6. Discussion

As the integration of AI into dermatology continues to evolve, ChatGPT stands out as a particularly promising tool. Unlike traditional AI systems that are primarily focused on image analysis and predictive modelling, ChatGPT utilizes natural language processing to enhance communication, patient education, and clinical decision-making within dermatology. This expanded functionality offers new possibilities for improving patient care, especially in teledermatology—a field that saw significant growth during the COVID-19 pandemic and continues to evolve with the incorporation of AI tools [51]. Given these advancements, it is crucial to explore the clinical implications and efficacy of ChatGPT in dermatology, particularly how it performs in real-world applications and patient interactions. Additionally, as ChatGPT becomes more integrated into education and clinical practice, it is essential to consider the ethical implications, such as data privacy, accuracy of information, and the potential for bias, to ensure responsible and equitable use in patient care.

The selection of ChatGPT for this study was driven by its public launch in November 2022 [1], which marked the beginning of its application in various fields, including healthcare and dermatology. By focusing on literature published from November 2022 to August 2024, we ensured that our review reflects the most recent developments following the launch of ChatGPT, capturing the latest advancements and discussions on its role in dermatology. Compared to other LLMs, ChatGPT’s superior performance in handling medical queries, particularly in dermatology, provides a strong foundation for understanding the potential and limitations of AI in this field. Furthermore, the ethical and legal considerations associated with AI use in dermatology are particularly relevant to ChatGPT, given its prominent role in the broader AI landscape. These factors collectively justify the focus on ChatGPT in this review, ensuring that the findings are both relevant and practically applicable to current clinical practices. Table 3 provides an overview of the various applications of ChatGPT in dermatology, including its benefits, key studies, and the challenges associated with its use in clinical practice.

### 6.1. Clinical Implications and Efficacy of ChatGPT

The clinical implications of ChatGPT in dermatology are significant, with several studies highlighting its potential. For example, research has demonstrated that ChatGPT can successfully pass dermatology specialty certificate examinations, positioning it as a useful tool for clinical decision support [52]. Moreover, ChatGPT has shown reliability across a variety of dermatological conditions, though it faces limitations in diagnosing complex cases like cutaneous neoplasms due to the subjective nature of physical exam descriptors and the variability of skin cancer presentations [23].

In patient interactions, ChatGPT has shown promising results in addressing common dermatological inquiries. Studies evaluating its responses to questions about conditions such as rosacea, melanoma, and basal cell carcinoma have found high levels of reliability and clinical applicability [20,21]. Additionally, ChatGPT’s ability to generate educational materials for patients, particularly for common dermatological conditions like acne, underscores its potential to enhance patient knowledge and engagement [39]. Despite these benefits, the possibility of ChatGPT providing incomplete or inaccurate information remains a concern, necessitating a cautious approach to its clinical application [55].

### 6.2. Ethical Challenges

AI’s role in dermatology, particularly through tools like ChatGPT, introduces significant ethical concerns. A scoping review by Gordon et al. (2024) highlights issues such as potential misdiagnosis, data security risks, and privacy violations that arise from the use of AI in clinical settings [23]. These concerns are particularly pressing when considering the impact of algorithmic bias, which can result in unequal care for patients, especially those with skin tones that are underrepresented in AI training datasets. The ability of ChatGPT to generate responses that may be factually incorrect or misleading—known as “hallucinations”—further complicates its ethical application in patient care [56]. Moreover, the erosion of the traditional physician–patient relationship is another ethical dilemma posed by AI. Overreliance on AI tools could diminish the humanistic aspects of patient care, leading to reduced empathy and trust between patients and healthcare providers. Transparency and the clear disclosure of AI-generated content are crucial to maintaining the integrity of medical practice and ensuring that patients remain informed and involved in their care decisions [57].

### 6.3. Legal Considerations

The legal landscape surrounding the use of AI in dermatology is still evolving, with significant implications for liability, data privacy, and regulatory oversight. Rosic (2024) discusses the complex legal challenges posed by AI in healthcare, particularly the unclear allocation of responsibility when AI-driven decisions lead to patient harm [53]. As AI tools like ChatGPT are increasingly used to assist in diagnosis and treatment planning, it becomes crucial to establish clear legal guidelines that define the extent of AI’s role and the corresponding liability of healthcare providers and AI developers [58]. Data privacy is another critical legal concern, especially given the sensitive nature of medical information. Chen (2024) emphasizes the importance of robust data privacy protections throughout the lifecycle of AI applications in healthcare [59]. The integration of LLMs into dermatology must adhere to stringent data protection standards to prevent unauthorized access and misuse of patient data, which could lead to significant legal and ethical repercussions.

### 6.4. Recommendations for Ethical and Legal Compliance

Bias in AI-generated content is a major ethical challenge, particularly in dermatology where the accuracy of diagnosis often depends on recognizing subtle differences in skin conditions across diverse populations. Singh et al. (2023) note that AI models trained on biased datasets are likely to perpetuate existing healthcare disparities, emphasizing the need for inclusive training data that represent a broad spectrum of skin types and conditions [60]. To address these ethical and legal challenges, it is essential to establish comprehensive guidelines and regulatory frameworks that ensure the responsible use of AI in dermatology [61,62]. This includes developing standards for AI transparency, bias mitigation, and data privacy protection. Furthermore, ongoing research and collaboration between AI developers, dermatologists, ethicists, and legal experts are necessary to refine these guidelines and adapt them to the rapidly evolving landscape of AI in healthcare. By proactively addressing these challenges, the dermatology community can harness the benefits of AI tools like ChatGPT while safeguarding patient trust and ensuring equitable, high-quality care. Table 4 provides an overview of the various applications of ChatGPT in dermatology, including its benefits, key studies, and the challenges associated with its use in clinical practice.

### 6.5. Future Directions and Research

ChatGPT has shown promise, such as in passing dermatology certification exams and addressing patient queries, but when it comes to image-based diagnostic tasks, deep learning algorithms, like convolutional neural networks (CNNs) and deep CNNs, have consistently outperformed it. These AI models have been widely applied in dermatology, achieving high diagnostic accuracy for conditions such as melanoma, with sensitivity and specificity rates often exceeding 70–75% [63,64]. A systematic review of 64 studies on deep learning in dermatology highlighted that, while these models are effective in diagnosing skin diseases, challenges remain, including limited generalizability, algorithmic bias, and inadequate reporting of participant demographics [65]. Most datasets used in these studies are drawn from Asian populations, offering limited representation of diverse skin types, such as Fitzpatrick types, and only 41% of the studies made their algorithm code publicly available, which hinders reproducibility [66]. Tools like ADAE, designed for melanoma classification, have significantly improved the accuracy of dermatologists in diagnosing suspicious lesions, showcasing the potential of AI in dermoscopic evaluations [67]. While ChatGPT’s strengths lie in patient education and clinical decision support, incorporating dermatoscopic image analysis into its framework or integrating it with AI-driven diagnostic tools like Dr. Dermbot could greatly enhance its utility in dermatology diagnostics.

The future of ChatGPT in dermatology is promising, but further research and development are needed to address these existing challenges and fully unlock its potential in image-based diagnostics. One critical area for improvement is the accuracy of AI-driven chatbots in distinguishing between benign and malignant skin lesions. While ChatGPT has shown potential in supporting differential diagnoses, its performance in handling complex cases, such as rare and genetic disorders, remains inconsistent [68]. To improve its clinical utility, further research should focus on enhancing diagnostic algorithms to better recognize subtle dermatological variations and integrate multimodal data, such as dermoscopy and histopathology results.

Another key area for future research is bias mitigation. Expanding and diversifying the datasets used to train LLM models are crucial for ensuring that ChatGPT can cater to a wide range of skin tones, ethnicities, and demographic factors [60]. Current models may underperform in populations that are underrepresented in the training data, which raises significant ethical concerns [69]. Future work should prioritize building more inclusive training datasets that represent the full diversity of dermatological presentations to reduce algorithmic bias and promote equitable healthcare outcomes.

The ethical and legal implications of using LLM models in dermatology also require further investigation [70]. As AI-driven chatbots become more integrated into clinical practice, it is essential to develop robust guidelines and ethical frameworks that address issues like data privacy, informed consent, and liability in AI-driven clinical decision-making. These frameworks should ensure that AI tools like ChatGPT complement human expertise rather than replace it, thereby maintaining the patient–provider relationship.

Collaborative efforts between AI developers, dermatologists, regulatory bodies, and ethicists would be key to ensuring responsible AI adoption [71]. Establishing regulatory standards for the deployment and monitoring of LLM models in clinical settings will help mitigate the risks associated with its use. Moreover, continuous oversight and periodic audits of AI tools in healthcare should be implemented to ensure compliance with evolving ethical and legal standards.

**Figure 2:**
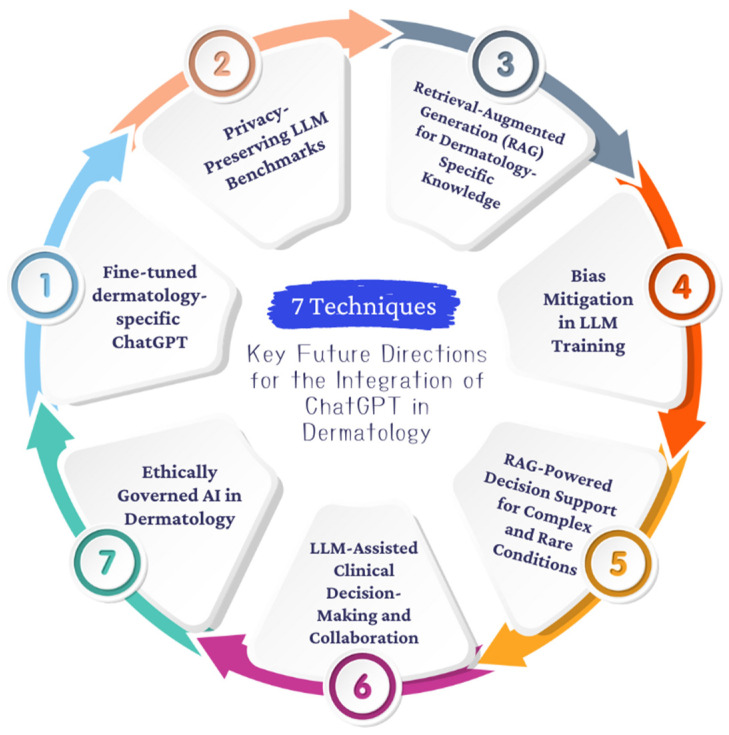
Key future directions for the integration of ChatGPT into dermatology.

In addition, further studies should explore the application of customized ChatGPT in specialized areas of dermatology, such as rare skin disorders, where diagnostic complexity often exceeds current AI capabilities. By refining ChatGPT’s ability to handle complex dermatological conditions, it can serve as a more robust tool in clinical practice [72,73]. To summarize, Figure 2 provides a roadmap for the key areas requiring further development, including improving diagnostic accuracy, mitigating algorithmic bias, expanding diverse datasets, and creating robust ethical and regulatory frameworks. Informed by a comprehensive literature review and expert input from the authors, this figure visually represents the roadmap for the future integration of ChatGPT into dermatological practice and the ongoing collaboration needed between AI researchers and healthcare professionals.

## 7. Conclusions

ChatGPT represents a promising development in dermatology, with the potential to enhance clinical decision-making, professional education, patient education and interaction, communication, and medical writing. However, its integration into dermatological practice must be approached with caution, particularly when it comes to tasks involving image-based diagnostics, where deep learning models like CNNs have shown superior performance. ChatGPT’s strengths lie in text-based applications, making it a valuable adjunct for patient interaction and support, but it cannot yet replace the critical expertise of dermatologists, especially in complex diagnostic scenarios.

Future research should focus on improving ChatGPT’s diagnostic capabilities by integrating it with AI models optimized for image recognition, diversifying training datasets to ensure better representation of skin types, and addressing ethical challenges like algorithmic bias and data privacy. Establishing clear clinical guidelines and regulatory frameworks will be essential to ensure that AI tools like ChatGPT are used responsibly, enhancing rather than replacing human expertise. Ultimately, while ChatGPT has the potential to revolutionize aspects of dermatological care, its success would depend on thoughtful integration and collaboration with clinical experts.

## Figures and Tables

**Table 1:** Summary of ChatGPT’s performance in dermatology certification examinations.

Study	Version of ChatGPT	Exam Type	Score Achieved	Pass Mark	Key Findings
Joly-Chevrier et al., 2023 [34]	ChatGPT-3.5	Practice Dermatology Board Certification Examination	59.3%	N/A	While ChatGPT-3.5 showed competence in basic science and pediatric dermatology sections, it struggled with more complex diagnostic categories, such as benign and malignant neoplasms. This reveals the limitations of earlier versions of ChatGPT in clinical applications that require nuanced understanding.
Passby et al., 2024 [35]	ChatGPT-4	Specialty Certificate Examination in Dermatology	90%	70–72%	ChatGPT-4 outperformed ChatGPT-3.5 with a significant margin, showcasing its improved understanding and application in dermatology-related questions. The AI demonstrated strong performance across various domains, indicating its potential utility in aiding dermatology education and certification.
Guillen-Grima et al., 2023 [36]	GPT-4	Spanish Medical Residency Entrance Examination	86.81%	N/A	GPT-4 demonstrated a broad understanding of medical queries across various specialties, including dermatology, indicating its enhanced capability over GPT-3.5. Its performance highlights the model’s growing proficiency in handling diverse and complex medical content.

**Table 2:** ChatGPT’s reliability and applicability in dermatological patient queries.

Study	Condition	Reliability (%)	Clinical Applicability (%)	Key Observations
Young et al., 2023 [20]	Melanoma	4.88/5 (Accuracy)	64% (Clinical Use)	ChatGPT provided accurate information suitable for general patient education on melanoma. However, its responses were not always sufficiently detailed for clinical decision-making, emphasizing the need for human oversight in complex cases.
Yan et al., 2024 [21]	Rosacea	92.22–97.78%	98.61–100.00%	ChatGPT demonstrated high reliability in answering patient questions about rosacea, with a strong alignment with clinical guidelines. The AI’s responses were found to be both accurate and practical, making it a valuable tool for patient education in chronic skin conditions.
Ferreira et al., 2023 [37]	Multiple Conditions	88% (Appropriate Responses)	N/A	ChatGPT provided appropriate responses to 88% of patient queries across various dermatological conditions. Inaccuracies were noted in complex topics.
Trager et al., 2023 [38]	Basal Cell Carcinoma	84% (Appropriate Responses)	N/A	ChatGPT provided appropriate responses to 84% of patient queries about BCC. Some responses contained inaccuracies that could impact patient outcomes.
Mondal et al., 2023 [39]	Acne	Readability: 46.94	N/A	The study found that ChatGPT could generate educational material for acne that was easily understandable for high school to early college students. However, the high text similarity index suggests a need for careful review to ensure originality and prevent redundancy.

**Table 3:** Overview of ChatGPT applications in dermatology: benefits, key studies, and challenges.

Application Area	Description	Key Benefits	Key Studies Referenced	Challenges
Clinical Decision Support	Assists in generating differential diagnoses and providing decision support for dermatological conditions.	Enhances diagnostic accuracy, particularly in straightforward cases, and aids clinicians in decision-making.	Yan et al., 2024 [21]; Lewandowski et al., 2024 [52]	Struggles with complex diagnoses, particularly in cases like cutaneous neoplasms.
Patient Education	Generates educational content on various dermatological conditions, providing patients with understandable information.	Improves patient knowledge and engagement, supports self-care and adherence to treatment plans.	Mondal et al., 2023 [39]	Risk of providing overly simplistic or generalized information.
Teledermatology	Supports remote patient consultations by providing initial assessments and guidance.	Increases access to dermatological care, especially in underserved and rural areas.	Ruggiero et al., 2022 [51]	Potential for misdiagnosis due to lack of visual or tactile examination.
Medical Writing Assistance	Assists in drafting and editing manuscripts, case reports, and educational materials.	Streamlines the writing process, allowing for clinicians to focus more on content and interpretation.	Potestio et al., 2024 [31]; Reynolds et al., 2024 [47]	Concerns over the originality and quality of AI-generated content.
Peer Review Process	Used to assist in the review of medical manuscripts by providing initial feedback and evaluation.	Enhances efficiency in the peer review process, reducing time burdens on human reviewers.	Khalifa and Ibrahim, 2024 [48]; Saad et al., 2024 [49]	Limited ability to replace human judgment in complex reviews.
Rural Healthcare Delivery	Provides timely medical advice in regions with limited access to dermatologists.	Bridges the healthcare gap in rural and underserved areas, offering initial guidance on dermatological issues.	Ahmed et al., 2023 [43]	Risk of misinformation due to lack of context and detailed examination.
Ethical and Legal Compliance	Monitors and ensures adherence to ethical standards and legal guidelines in dermatological practice.	Promotes responsible use of AI in dermatology, ensuring patient safety and data privacy.	Gordon et al., 2024 [23]; Rosic, 2024 [53]; Lambert and Grzybowski, 2024 [54]	Challenges in defining legal responsibility and managing data security.

**Table 4:** Ethical and legal challenges associated with AI integration into dermatology.

Aspect	Challenge	Key Studies Referenced	Proposed Solutions
Data Privacy	Risk of data breaches and unauthorized access	Chen, 2024 [59]	Implementation of stringent data privacy policies and secure data handling protocols throughout the AI lifecycle to protect sensitive patient information.
Algorithmic Bias	Disparities in care due to underrepresented skin tones	Singh et al., 2023 [60]; Gordon et al., 2024 [23]	Ensuring diversity in AI training datasets by including a broad spectrum of skin types and conditions to mitigate bias and ensure equitable care.
Legal Liability	Unclear responsibility when AI-driven decisions cause harm	Wang et al., 2023 [56]; Rosic, 2024 [53]	Establishment of clear legal guidelines that define the role and liability of AI in clinical decision-making, ensuring accountability among healthcare providers and AI developers.
Ethical Concerns	Hallucinations leading to misinformation	Grzybowski et al., 2024 [29]; Lambert and Grzybowski, 2024 [54]	Development of transparency protocols, regular audits, and AI content disclosure to prevent the spread of misinformation and maintain trust in AI-assisted care.

## References

[1] OpenAI (2022). ChatGPT—Release Notes. https://help.openai.com/en/articles/6783457-chatgpt-release-notes.

[2] Zhang Z., Zhang J., Duan L., Tan C. (2024). ChatGPT in Dermatology: Exploring the Limited Utility Amidst the Tech Hype. Front. Med..

[3] Roumeliotis K.I., Tselikas N.D. (2023). ChatGPT and OpenAI models: A preliminary review. Future Internet.

[4] Goktas P., Karakaya G., Kalyoncu A.F., Damadoglu E. (2023). Artificial Intelligence Chatbots in Allergy and Immunology Practice: Where Have We Been and Where Are We Going?. J. Allergy Clin. Immunol. Pract..

[5] Goktas P., Kucukkaya A., Karacay P. (2023). Leveraging the Efficiency and Transparency of Artificial Intelligence-Driven Visual Chatbot through Smart Prompt Learning Concept. Skin Res. Technol..

[6] Khan S.S., Polo Silveira L., Varma A., Maurer T. (2024). The Ethical and Legal Considerations for the Use of Artificial Intelligence in Global Health Dermatology. Clin. Exp. Dermatol..

[7] Alessandri-Bonetti M., Liu H.Y., Giorgino R., Nguyen V.T., Egro F.M. (2024). The First Months of Life of ChatGPT and Its Impact in Healthcare: A Bibliometric Analysis of the Current Literature. Ann. Biomed. Eng..

[8] Gui H., Omiye J.A., Chang C.T., Daneshjou R. (2024). The Promises and Perils of Foundation Models in Dermatology. J. Investig. Dermatol..

[9] Huang Y., Tang K., Chen M. (2024). A Comprehensive Survey on Evaluating Large Language Model Applications in the Medical Industry. arXiv.

[10] Mu X., Lim B., Seth I., Xie Y., Cevik J., Sofiadellis F., Rozen W.M. (2024). Comparison of Large Language Models in Management Advice for Melanoma: Google’s AI BARD, BingAI and ChatGPT. Skin Health Dis..

[11] Sandmann S., Riepenhausen S., Plagwitz L., Varghese J. (2024). Systematic Analysis of ChatGPT, Google Search and Llama 2 for Clinical Decision Support Tasks. Nat. Commun..

[12] Raiaan M.A.K., Mukta M.S.H., Fatema K., Fahad N.M., Sakib S., Mim M.M.J., Azam S. (2024). A Review on Large Language Models: Architectures, Applications, Taxonomies, Open Issues and Challenges. IEEE Access.

[13] Liu X., Duan C., Kim M.K., Zhang L., Jee E., Maharjan B., Huang Y., Du D., Jiang X. (2024). Claude 3 Opus and ChatGPT with GPT-4 in Dermoscopic Image Analysis for Melanoma Diagnosis: Comparative Performance Analysis. JMIR Med. Inform..

[14] Luo N., Zhong X., Su L., Cheng Z., Ma W., Hao P. (2023). Artificial Intelligence-Assisted Dermatology Diagnosis: From Unimodal to Multimodal. Comput. Biol. Med..

[15] Strzelecki M., Kociołek M., Strąkowska M., Kozłowski M., Grzybowski A., Szczypiński P.M. (2024). Artificial Intelligence in the Detection of Skin Cancer: State of the Art. Clin. Dermatol..

[16] Goktas P., Gülseren D., Tobin A.M. (2024). Large Language and Vision Assistant in Dermatology: A Game Changer or Just Hype?. Clin. Exp. Dermatol..

[17] Sengupta D. (2024). Artificial Intelligence in Diagnostic Dermatology: Challenges and the Way Forward. Indian Dermatol. Online J..

[18] Dunn C., Hunter J., Steffes W., Whitney Z., Foss M., Mammino J., Leavitt A., Hawkins S.D., Dane A., Yungmann M. (2023). Artificial Intelligence-Derived Dermatology Case Reports Are Indistinguishable from Those Written by Humans: A Single-Blinded Observer Study. J. Am. Acad. Dermatol..

[19] Shapiro J., Lyakhovitsky A. (2024). Revolutionizing Teledermatology: Exploring the Integration of AI, Including GPT Chatbots for AI-Driven Anamnesis, Diagnosis, and Treatment Plans. Clin. Dermatol..

[20] Young J.N., O’Hagan R., Poplausky D., Levoska M.A., Gulati N., Ungar B., Ungar J. (2023). The Utility of ChatGPT in Generating Patient-Facing and Clinical Responses for Melanoma. J. Am. Acad. Dermatol..

[21] Yan S., Du D., Liu X., Dai Y., Kim M.K., Zhou X., Jiang X. (2024). Assessment of the Reliability and Clinical Applicability of ChatGPT’s Responses to Patients’ Common Queries About Rosacea. Patient Prefer. Adherence.

[22] Rundle C.W., Szeto M.D., Presley C.L., Shahwan K.T., Carr D.R. (2024). Analysis of ChatGPT Generated Differential Diagnoses in Response to Physical Exam Findings for Benign and Malignant Cutaneous Neoplasms. J. Am. Acad. Dermatol..

[23] Gordon E.R., Trager M.H., Kontos D., Weng C., Geskin L.J., Dugdale L.S., Samie F.H. (2024). Ethical Considerations for Artificial Intelligence in Dermatology: A Scoping Review. Br. J. Dermatol..

[24] Smith P., Johnson C.E., Haran K., Orcales F., Kranyak A., Bhutani T., Riera-Monroig J., Liao W. (2024). Advancing Psoriasis Care through Artificial Intelligence: A Comprehensive Review. Curr. Dermatol. Rep..

[25] Pillai A., Parappally-Joseph S., Hardin J. (2024). Evaluating the Diagnostic and Treatment Recommendation Capabilities of GPT-4 Vision in Dermatology. medRxiv.

[26] Shifai N., van Doorn R., Malvehy J., Sangers T.E. (2024). Can ChatGPT Vision Diagnose Melanoma? An Exploratory Diagnostic Accuracy Study. J. Clin. Med..

[27] Gomolin A., Netchiporouk E., Gniadecki R., Litvinov I.V. (2020). Artificial Intelligence Applications in Dermatology: Where Do We Stand?. Front. Med..

[28] Beltrami E.J., Grant-Kels J.M. (2023). Dermatology in the Wake of an AI Revolution: Who Gets a Say?. J. Am. Acad. Dermatol..

[29] Grzybowski A., Jin K., Wu H. (2024). Challenges of Artificial Intelligence in Medicine and Dermatology. Clin. Dermatol..

[30] Joseph J., Chettyparambil Lalchand T. (2024). The Synergy of Skin and Science—A Comprehensive Review of Artificial Intelligence’s Impact on Dermatology. CosmoDerma.

[31] Potestio L., Megna M., Cacciapuoti S., Martora F., Villani A. (2024). ChatGPT and Medical Writing in Dermatology: Why Should We Keep Writing?. Clin. Exp. Dermatol..

[32] Rubeta N.M. (2024). Widening the Scope of Artificial Intelligence Applications in Dermatology. Clin. Exp. Dermatol..

[33] Giansanti D. (2023). The Artificial Intelligence in Teledermatology: A Narrative Review on Opportunities, Perspectives, and Bottlenecks. Int. J. Environ. Res. Public Health.

[34] Joly-Chevrier M., Nguyen A.X.-L., Lesko-Krleza M., Lefrançois P. (2023). Performance of ChatGPT on a Practice Dermatology Board Certification Examination. J. Cutan. Med. Surg..

[35] Passby L., Jenko N., Wernham A. (2024). Performance of ChatGPT on Specialty Certificate Examination in Dermatology Multiple-Choice Questions. Clin. Exp. Dermatol..

[36] Guillen-Grima F., Guillen-Aguinaga S., Guillen-Aguinaga L., Alas-Brun R., Onambele L., Ortega W., Montejo R., Aguinaga-Ontoso E., Barach P., Aguinaga-Ontoso I. (2023). Evaluating the Efficacy of ChatGPT in Navigating the Spanish Medical Residency Entrance Examination (MIR): Promising Horizons for AI in Clinical Medicine. Clin. Pract..

[37] Ferreira A.L., Chu B., Grant-Kels J.M., Ogunleye T., Lipoff J.B. (2023). Evaluation of ChatGPT Dermatology Responses to Common Patient Queries. JMIR Dermatol..

[38] Trager M.H., Queen D., Bordone L.A., Geskin L.J., Samie F.H. (2023). Assessing ChatGPT Responses to Common Patient Queries Regarding Basal Cell Carcinoma. Arch. Dermatol. Res..

[39] Mondal H., Mondal S., Podder I. (2023). Using ChatGPT for Writing Articles for Patients’ Education for Dermatological Diseases: A Pilot Study. Indian Dermatol. Online J..

[40] Lent H.C., Ortner V.K., Karmisholt K.E., Wiegell S.R., Nissen C.V., Omland S.H., Haedersdal M. (2024). A Chat About Actinic Keratosis: Examining Capabilities and User Experience of ChatGPT as a Digital Health Technology in Dermato-Oncology. JEADV Clin. Pract..

[41] Wilhelm T.I., Roos J., Kaczmarczyk R. (2023). Large Language Models for Therapy Recommendations Across 3 Clinical Specialties: Comparative Study. J. Med. Internet Res..

[42] Ayub I., Hamann D., Hamann C.R., Davis M.J. (2023). Exploring the Potential and Limitations of Chat Generative Pre-Trained Transformer (ChatGPT) in Generating Board-Style Dermatology Questions: A Qualitative Analysis. Cureus.

[43] Ahmed S.K., Hussein S., Aziz T.A., Chakraborty S., Islam M.R., Dhama K. (2023). The Power of ChatGPT in Revolutionizing Rural Healthcare Delivery. Health Sci. Rep..

[44] Eysenbach G. (2023). The Role of ChatGPT, Generative Language Models, and Artificial Intelligence in Medical Education: A Conversation with ChatGPT and a Call for Papers. JMIR Med. Educ..

[45] Garg R.K., Urs V.L., Agarwal A.A., Chaudhary S.K., Paliwal V., Kar S.K. (2023). Exploring the Role of ChatGPT in Patient Care (Diagnosis and Treatment) and Medical Research: A Systematic Review. Health Promot. Perspect..

[46] Tan S., Xin X., Wu D. (2024). ChatGPT in Medicine: Prospects and Challenges: A Review Article. Int. J. Surg..

[47] Reynolds K., Nadelman D., Durgin J., Ansah-Addo S., Cole D., Fayne R., Harrell J., Ratycz M., Runge M., Shepard-Hayes A. (2024). Comparing the Quality of ChatGPT- and Physician-Generated Responses to Patients’ Dermatology Questions in the Electronic Medical Record. Clin. Exp. Dermatol..

[48] Khalifa A.A., Ibrahim M.A. (2024). Artificial Intelligence (AI) and ChatGPT Involvement in Scientific and Medical Writing, a New Concern for Researchers: A Scoping Review. Arab. Gulf J. Sci. Res..

[49] Saad A., Jenko N., Ariyaratne S., Birch N., Iyengar K.P., Davies A.M., Botchu R. (2024). Exploring the Potential of ChatGPT in the Peer Review Process: An Observational Study. Diabetes Metab. Syndr. Clin. Res. Rev..

[50] Stade E.C., Stirman S.W., Ungar L.H., Boland C.L., Schwartz H.A., Yaden D.B., Eichstaedt J.C. (2024). Large Language Models Could Change the Future of Behavioral Healthcare: A Proposal for Responsible Development and Evaluation. NPJ Ment. Health Res..

[51] Ruggiero A., Martora F., Fabbrocini G., Villani A., Marasca C., Megna M., Fornaro L., Comune R., Potestio L. (2022). The Role of Teledermatology during the COVID-19 Pandemic: A Narrative Review. Clin. Cosmet. Investig. Dermatol..

[52] Lewandowski M., Łukowicz P., Świetlik D., Barańska-Rybak W. (2024). ChatGPT-3.5 and ChatGPT-4 Dermatological Knowledge Level Based on the Specialty Certificate Examination in Dermatology. Clin. Exp. Dermatol..

[53] Rosic A. (2024). Legal Implications of AI in Healthcare. Clin. Dermatol..

[54] Lambert W.C., Grzybowski A. (2024). Dermatology and Artificial Intelligence. Clin. Dermatol..

[55] Wong R.S.Y., Ming L.C., Ali R.A.R. (2023). The Intersection of ChatGPT, Clinical Medicine, and Medical Education. JMIR Med. Educ..

[56] Wang C., Liu S., Yang H., Guo J., Wu Y., Liu J. (2023). Ethical Considerations of Using ChatGPT in Health Care. J. Med. Internet Res..

[57] Sallam M. (2023). ChatGPT Utility in Healthcare Education, Research, and Practice: Systematic Review on the Promising Perspectives and Valid Concerns. MDPI Healthc..

[58] Alvarez J.M., Colmenarejo A.B., Elobaid A., Fabbrizzi S., Fahimi M., Ferrara A., Ghodsi S., Mougan C., Papageorgiou I., Reyero P. (2024). Policy Advice and Best Practices on Bias and Fairness in AI. Ethics Inf. Technol..

[59] Chen S. (2024). Potential Applications and Safety of Large Language Models in Healthcare. Interdiscip. Humanit. Commun. Stud..

[60] Singh J., Sillerud B., Singh A. (2023). Artificial Intelligence, Chatbots and ChatGPT in Healthcare—Narrative Review of Historical Evolution, Current Application, and Change Management Approach to Increase Adoption. J. Med. Artif. Intell..

[61] Evans M., White L., Sinclair M. (2023). The EU’s AI Act: The Position Is Agreed. Data Prot. Rep..

[62] European Parliament (2024). EU AI Act: First Regulation on Artificial Intelligence. European Parliament News..

[63] Brinker T.J., Hekler A., Enk A.H., Berking C., Haferkamp S., Hauschild A., Weichenthal M., Klode J., Schadendorf D., Holland-Letz T. (2019). Deep Neural Networks are Superior to Dermatologists in Melanoma Image Classification. Eur. J. Cancer.

[64] Brinker T.J., Hekler A., Enk A.H., Klode J., Hauschild A., Berking C., Schilling B., Haferkamp S., Schadendorf D., Fröhling S. (2019). A Convolutional Neural Network Trained with Dermoscopic Images Performed on Par with 145 Dermatologists in a Clinical Melanoma Image Classification Task. Eur. J. Cancer.

[65] Du-Harpur X., Watt F.M., Luscombe N.M., Lynch M.D. (2020). What is AI? Applications of Artificial Intelligence to Dermatology. Br. J. Dermatol..

[66] LeCun Y., Bengio Y., Hinton G. (2015). Deep Learning. Nature.

[67] Marchetti M.A., Cowen E.A., Kurtansky N.R., Weber J., Dauscher M., DeFazio J., Deng L., Dusza S.W., Haliasos H., Halpern A.C. (2023). Prospective Validation of Dermoscopy-based Open-Source Artificial Intelligence for Melanoma Diagnosis (PROVE-AI study). NPJ Digit. Med..

[68] Zampatti S., Peconi C., Megalizzi D., Calvino G., Trastulli G., Cascella R., Strafella C., Caltagirone C., Giardina E. (2024). Innovations in Medicine: Exploring ChatGPT’s Impact on Rare Disorder Management. Genes.

[69] Yang J., Wang Z., Lin Y., Zhao Z. (2024). Global Data Constraints: Ethical and Effectiveness Challenges in Large Language Model. arXiv.

[70] Ong J.C.L., Chang S.Y.H., William W., Butte A.J., Shah N.H., Chew L.S.T., Liu N., Doshi-Velez F., Lu W., Savulescu J. (2024). Ethical and Regulatory Challenges of Large Language Models in Medicine. Lancet Digit. Health.

[71] Bouderhem R. (2024). Shaping the Future of AI in Healthcare through Ethics and Governance. Humanit. Soc. Sci. Commun..

[72] Arslan B., Eyupoglu G., Korkut S., Turkdogan K.A., Altinbilek E. (2024). The Accuracy of AI-Assisted Chatbots on the Annual Assessment Test for Emergency Medicine Residents. J. Med. Surg. Public Health.

[73] Triantafyllopoulos L., Feretzakis G., Tzelves L., Sakagianni A., Verykios V.S., Kalles D. (2024). Evaluating the Interactions of Medical Doctors with Chatbots Based on Large Language Models: Insights from a Nationwide Study in the Greek Healthcare Sector Using ChatGPT. Comput. Hum. Behav..

